# The Influence of Non-Tobacco Nicotine Dependence on Wound Healing and Postoperative Outcomes in Body Contouring Surgery: A Propensity-Score Matched Analysis

**DOI:** 10.1093/asjof/ojag138.009

**Published:** 2026-07-24

## Abstract

**Purpose:**

The popularity of electronic cigarettes and other non-tobacco nicotine delivery systems has grown rapidly, yet their implications for surgical outcomes remain uncertain. Unlike combustible tobacco, which has long been linked to impaired wound healing and postoperative complications, the risks associated with isolated nicotine exposure are understudied despite both containing the same biologically active components that impair tissue repair. Body contouring procedures, which often involve extensive soft tissue undermining and rely heavily on vascular integrity for wound healing, may be particularly susceptible to the effects of nicotine. This study aims to investigate the association between non-tobacco nicotine dependence (NTND) and postoperative complications following body contouring surgery using a large multicenter electronic health record database.

**Methods:**

A retrospective cohort study was conducted using the TriNetX Research Network. Adult patients who underwent body contouring procedures, including panniculectomy, abdominoplasty, liposuction, autologous fat grafting, mastopexy, thighplasty, brachioplasty, and other areas of excessive skin excision, were identified based on relevant CPT codes. Patients were divided into two cohorts based on the presence of documented NTND within one year prior to surgery, compared with patients without any form of nicotine dependence. Propensity score matching (1:1) was performed to balance demographic and clinical variables including age, race, body mass index, hypertension, diabetes mellitus, history of chemotherapy, and use of anticoagulant or antiplatelet therapy. Postoperative outcomes were evaluated cumulatively at 30 days, 90 days, and 1 year, including any surgical site complication, wound-related complications (e.g. disruption, seroma, hematoma, or necrosis), wound dehiscence, delayed healing, infection, postoperative bleeding (e.g., hematoma, hemorrhage), and return to the emergency department or hospital readmission. Each subsequent interval included complications from prior timepoints to ensure complete event capture, as TriNetX suppresses outcomes with fewer than ten events. Risk ratios with 95% confidence intervals were calculated for each outcome, and statistical significance was defined as p < 0.05.

**Results:**

Before matching, a total of 93,572 patients were identified, including 1,671 with NTND and 91,901 controls. Following 1:1 propensity score matching, 1,617 patients remained in each cohort for outcome comparison. At one year, patients with NTND demonstrated significantly higher rates of postoperative complications across multiple domains. Compared with matched controls, NTND patients experienced higher rates of any surgical site complication (15.43% vs. 8.94%; RR 1.73, 95% CI 1.38– 2.16; p<0.0001), wound-related complications (7.08% vs. 4.58%; RR 1.55, 95% CI 1.15–2.08; p=0.0036), wound dehiscence or delayed healing (5.32% vs. 2.89%; RR 1.84, 95% CI 1.29–2.64; p=0.0006), infection (7.54% vs. 5.29%; RR 1.42, 95% CI 1.06–1.92; p=0.0193), and postoperative bleeding (4.69% vs. 3.12%; RR 1.51, 95% CI 1.05–2.16; p=0.0247). Rates of seroma formation and return to the emergency department or hospital readmission were not significantly different between cohorts. Findings at 30 and 90 days followed a similar pattern, with NTND patients demonstrating consistently higher absolute complication rates across all outcomes. Although several early differences did not reach statistical significance at shorter follow-up intervals, the direction and magnitude of risk were maintained, indicating a sustained adverse effect of nicotine exposure on wound healing over time.

**Conclusion:**

NTND was associated with significantly higher rates of surgical site, wound-related, and infectious complications following body contouring procedures. These findings suggest that nicotine exposure, even in the absence of combustible tobacco use, may impair wound healing and increase postoperative morbidity in procedures requiring large tissue surface area management and vascular perfusion. While early postoperative outcomes trended similarly, complications were most pronounced at one year, highlighting the persistent impact of nicotine on long-term healing. Because this analysis reflects associations rather than causation, further prospective and mechanistic studies are needed to investigate the biologic effects of non-tobacco nicotine exposure and guide perioperative management strategies.

